# Academy of Stomatology

**Published:** 1896-03

**Authors:** George D. B. Darby

**Affiliations:** Secretary


### ACADEMY OF STOMATOLOGY.

The regular meeting of the Academy was held December 17, 1895. In the absence of the President, Dr. James Truman, Vice-President, called the meeting to order.

Dr. Kirk on behalf of the Council made a report.

Dr. Younger was called upon to explain the workings of a dental society in San Francisco of which he was a member, and said, in response to the request, "The Stomatological Club was organized as a dental club for the purpose of entertaining members of the World's Fair Dental Congress. It had a membership reaching from China, the Hawaiian Islands, Chile, and Denver. We had also present several members from the East. After the Congress we were so impressed with the clinical work and the advantages that we determined then to maintain the Club, both for the social and the clinical features, which latter had become *the* feature of the Club, and for that purpose we meet every Tuesday afternoon at three o'clock. A clinician is appointed, and if he fails to do his duty he is fined ten dollars. At first, from timidity, the members preferred to pay the fine rather than perform the duties of the clinic, but they have all been so enthused with the advantages that there are in fact two or three ready all the time to take the place of any one who fails.

"It has become so well known that people come from a distance, and we have very important consultations. We found the name Dental Club did not express its use, so we called it the Stomatological Club. We hold clinics there as long as there is any light, and then adjourn for dinner and come back at half-past seven or eight o'clock, and every one must speak when called upon. We have a museum and library, and some valuable additions have been made. It has social and clinical features, but the clinical is paramount to everything. The cost of admission is twenty dollars, with dues three dollars a month.

"We have three rooms on Union Square, and have a degree which is conferred upon those who have merited it. In the last year ninety per cent. of the attendance acquired it; ninety per cent. gave clinics. There were sixteen who attained the degree of F.S.C. and they were awarded the Fellowship degree. The post-graduate course or degree is the Fellowship, so that every one of the sixteen is a Fellow of the Stomatological Club of California, with the right to sign himself F.S.C.

"The membership is restricted to thirty, but out of that we have had an average attendance of twenty-five every Tuesday. These men give up their business. They come from Oakland, where they must leave the office at two o'clock, and San José, which requires them to leave the office in the morning, to arrive at San Francisco at three o'clock, and you can understand the enthusiasm of the sixteen who have received their parchment; not a single one has resigned from the club, so it is not the possession of the parchment, but the love of learning and their fellows which constitutes the great attraction."

DISCUSSION.

*Dr. Burchard.*—In consideration of the importance of the subject brought up by the report from the Council, touching some of the points that Dr. Younger spoke of in regard to the Stomatological Club of San Francisco, I move that we hold a special meeting for the discussion of these matters on the first Tuesday of January. It is a subject of importance, involving a change in the constitution, and I think we should hold a special meeting to discuss it.

*Dr. Darby.*—In seconding that motion, which I do most heartily, I would say there are many things we should talk over, and we have no time to do it to-night. It is the general feeling among those with whom I have talked that we want to make this Academy as useful as possible; we should have the room occupied more than it has been in the past. The object in securing these rooms was to have a place where the members could come and obtain information, and with that view we decided to have clinics. I feel there is no reason why the rooms should not be occupied at least two or three days each month, where information can be given and exchanged.

There are many who want to come into the society who are debarred unless they conform to conditions which seem to them almost insurmountable. There are men already in the society who would be glad to give clinics; they are too retiring or modest to present papers, but they would give practical exhibits, and I heartily second the motion to meet the first Tuesday in January in order to discuss this whole subject.

Motion carried.

Dr. Kirk stated that the report of Council was simply its recommendation that the matter be considered by the society, and that it was so embodied in the report; that it was in no sense a ruling on the matter; simply a recommendation that the society consider the questions referred to.

Dr. Louis Jack, who was then called upon to read the paper of the evening, said, in introducing the subject,—

When some one suggested that the implantation of teeth would be a useful one to bring before this Academy, I agreed to make some presentation of it in order to facilitate discussion. I presumed that what I might have to say would be very short indeed, merely covering the general points of the subject, but I found, after I entered upon it, that it would be better to go over the whole field, omitting some details. I have dealt with it in a very simple and elementary manner, and have avoided as much of the technical language as possible, so that it would be comprehended by students as well as by ourselves.

(For Dr. Jack's paper, see page 133.)

Dr. Younger of San Francisco was called upon to open the discussion, and in response to the call, said,—

Mr. President and Gentlemen,—Somebody asked me a year or two ago if I had abandoned the implantation of teeth, and the reason he gave was that he had heard nothing from me for a number of years. I said the reason I had not written about it was that I had given it to the profession years ago, and was simply now waiting for time to show the operation as successful.

So far, my first cases are all intact. The first case I had was a young maiden, an Italian. This, to my surprise, remained in eighteen months. All her friends came up and shook the tooth and found it had become as firm as the others.

My next case was the 17th of August, succeeding. I planted five teeth, two of them on that day, the 17th, one a week later, one about the 1st of December, and the fifth one, a molar, on the year following. So that leaves four teeth that have been in four years, one over ten years, and one ten years about the first of next August, and they are all in perfect condition. A year or two ago, Dr. Daboll, of Paris, saw them, and he could not determine the teeth, or distinguish these from the original teeth.

There are quite a number of other cases that are from nine to ten years of age that are in excellent condition.

A week or so before I left, a lady visited my office and complained that the one I planted nine years ago was loose. I examined it and found what I supposed was a case of resorption of the root, so I made an appointment, and prepared a tooth for a case of transplantation; when I started in to remove it I found to my surprise a solid tooth. The one that had been planted nine years last April was in perfect condition. This shows that the operation is a permanent one; that is, if an operation will last ten years, you may look upon it as permanent. It promises in the case I have mentioned to last a lifetime, because there is nothing to indicate in appearance that there has been any absorption at all.

In regard to the operation, I would like to ask Dr. Jack how he makes his incision, because I think that is important in securing an artistic effect?

*Dr. Jack.*—I generally make the incision with the circular knife.

*Dr. Younger.*—In case there would be much absorption, that will give an unartistic appearance. I would like to show how I perform the operation to get an artistic result.

A lady went to New York for whom I planted four teeth. I sent her to a gentleman there who had performed this work a great deal and who makes a specialty of it, and I followed in three weeks. He said, "How do you do it? These teeth have been in six weeks, and I couldn't tell which of them you had planted." I said, "The thing ought to be done artistically." We want to resemble the teeth that grew there, otherwise I do not consider it artistic.

The majority of the profession look upon it as a thing to be dreaded. After the tooth has been thoroughly cleansed and all the substance removed, it is incapable of carrying infection. The only bacteria we have to dread is what is known as surgical bacteria. We don't care for the tubercular or syphilitic bacteria. Before performing the operation the mouth should be thoroughly sterilized, even the tissues underneath the gum, with a delicate syringe, and the person should gargle his throat; if possible, make the territory of the operation perfectly inhospitable to pathogenic germs; then, of course, the instruments should be sterilized; the hands should be thoroughly sterilized, and the finger-nails especially, to see that there is no dirt; remove this with soap and hot water and then sterilize them. With all these precautions it is impossible for bacteria to infect the parts. Then after the operation every two hours the patient should sterilize her mouth, and it should be kept antiseptic as long as the wound is open. I think it is due to these precautions that I have met with so much success.

The doctor then proceeded to illustrate his operation by blackboard drawings, explaining the cuts as he made them.

*Dr. Darby.*—Do you inject cocaine into the gum, or etherize the patient?

*Dr. Younger.*—I inject cocaine.

*Dr. Darby.*—I don't know that I have any desire to relate my own experience, but I want to bear testimony to four teeth I saw in a mouth that Dr. Younger planted at least eight years ago. There were two centrals and two bicuspids, and had been in eight years. With one of the centrals resorption had taken place, and I was forced to replant another tooth in its place. His remarks in regard to artistic appearance I can fully verify: the appearance was certainly beautiful, and there was no recession of the gum present.

*Dr. Younger.*—A gentleman in the Confederate service, who had in a charge lost three centrals and a lateral, came to me for advice five years ago. He had no alveolus except at the very end of the root. In this same gentleman one of the laterals I implanted had two failures; with the third I had excellent success.

I had a case where a lady came to me to have a tooth implanted, a right superior bicuspid; this was nine years ago, and I defy any dentist to select that tooth by observation or by resonance. She had one tooth out, the second superior bicuspid, and another one was diseased at the root, and she decided to have that one out and one implanted, and I performed this operation four times before I succeeded.

*Dr. Darby.*—What do you think is the form of attachment between the tooth and the alveolus?

*Dr. Younger.*—I think there is a great deal of what I understand to be physiological tissue and pericementum.

The doctor was asked, How long do you leave your appliances on before you consider it a success or failure?

*Dr. Younger.*—In the course of a month or two months, if the tooth does not take hold, I take it out and put in another. If there is no attachment, of course it is a failure ; if there is attachment to the gum and the tooth simply remains loose, I consider that a success.

Dr. Jack was called upon to close the discussion, and in doing so, said,—

I have nothing further to say, except that I will open the cases I have here.

Here is a little box with a number of splints in it, and I have two teeth ready for implantation. After I have prepared my case I keep them in a disinfecting solution and cover them until the operation is to be performed. This case has been prepared two or three weeks. The splint is to be wired fast to the tooth ; there is an arrangement of the wire which is cemented fast. You will readily see that that form of splint makes a secure attachment in all directions.

Here is one prepared to take the place of a tooth lost by pyorrhœa, a bicuspid tooth.

The doctor referred to one or two other specimens and passed them around among the members for inspection.

*Dr. Deane.*—Before Dr. Jack closes, I want to bear testimony to a few of the cases that he kindly invited me to his office to see, and one point that struck me is his method of retaining the tooth in the mouth. It would seem almost impossible for him to fail with the retaining fixture in place. I saw one case where he had just removed the fixture, and it was impossible to produce any movement of the tooth, and that was a case in which considerable pyorrhœa had existed. An additional cause of trouble was that pyorrhœa extended throughout the mouth, and yet this tooth was immovable and thoroughly resonant; I could not tell which tooth had been implanted.

*Dr. McQuillan.*—Dr. Jack kindly sent one of his patients over to me, and I was delighted with the result; particularly in one case where there had been considerable pyorrhœa.

*Dr. Jack.*—When I secure suitable teeth, I preserve them in a mixture of glycerin and water, equal parts, in order to keep them moist.

Dr. Register then gave a black-board illustration of an appliance for increasing the power of the dental engine, which, in whatever position it was held, gave an unvarying result. The arm of the engine to which the appliance was attached was exhibited to the society.

Dr. Darby then called the attention of those present to the fact that a collation had been prepared and that all were invited to partake of it.

Adjourned.

George D. B. Darby,
*Secretary.*

